# Supplementary material for: Phylogenetic correlations can suffice to infer protein partners from sequences
Source: PLoS Comput Biol. 2019 Oct 14;15(10):e1007179. doi: 10.1371/journal.pcbi.1007179 (PMC6812855; doi:10.1371/journal.pcbi.1007179)
Supplement: S1 Table — For each pair of E. coli proteins, we applied the original Mirrortree and pMirrortree methods, as described in Refs. [31, 34] and [35], respectively. The resulting scores quantify the similarity of the phylogenetic trees constructed from the orthologs of the two proteins considered. Note that paralogs are not included in this analysis, in contrast to the one presented in our paper. The Mirrortree score is a Pearson correlation between the sets of distances of the two protein families considered, and a large value is indicative of coevolution [31, 34]. Among the five pairs considered, LOLC-MACA and ACRE-ENVR have the smallest Mirrortree scores, in agreement with the fact that they are the only pairs that possess no known direct physical interactions. The pMirrortree score is a p-value that provides an assessment of the confidence in the tree similarity scores: a small pMirrortree score is indicative of coevolution [35]. Considering the standard significance threshold 0.05, BASS-BASR (HK-RR) and XDHA-XDHC have significant coevolution, while LOLC-MACA and ACRE-ENVR do not. This is in good agreement with the fact that BASS-BASR and XDHA-XDHC possess known direct physical interactions, while LOLC-MACA and ACRE-ENVR do not. Surprisingly, MALG-MALK has a large pMirrortree score while possessing a known direct physical interaction, but this is mitigated by the fact that the close paralogs POTI-POTA have significant coevolution according to their pMirrortree score. (PDF) [file pcbi.1007179.s008.pdf]

S1 Table for *Phylogenetic correlations can suffice to infer protein partners from sequences* by Guillaume Marmier, Martin Weigt and Anne-Florence Bitbol

Protein 1	Protein 2	Mirrortree score (Pearson correlation) [1, 2]	pMirrortree score (p-value) [3]	Comments
LOLC	MACA	0.82	0.76	
ACRE	ENVR	0.82	0.24	pMirrortree score was calculated between ACRE and ACRR (close paralog of ENVR).
BASS (HK)	BASR (RR)	0.96	0.003	
MALG	MALK	0.85	0.84	pMirrortree score is 0.013 when considering the close paralogs POTI-POTA of MALG-MALK.
XDHA	XDHC	0.86	0.002	

Mirrortree results for the protein pairs considered in Fig 6. For each pair of *E. coli* proteins, we applied the original Mirrortree and pMirrortree methods, as described in Refs. [1, 2] and [3], respectively. The resulting scores quantify the similarity of the phylogenetic trees constructed from the orthologs of the two proteins considered. Note that paralogs are not included in this analysis, in contrast to the one presented in our paper. The Mirrortree score is a Pearson correlation between the sets of distances of the two protein families considered, and a large value is indicative of coevolution [1, 2]. Among the five pairs considered, LOLC-MACA and ACRE-ENVR have the smallest Mirrortree scores, in agreement with the fact that they are the only pairs that possess no known direct physical interactions. The pMirrortree score is a p-value that provides an assessment of the confidence in the tree similarity scores: a small pMirrortree score is indicative of coevolution [3]. Considering the standard significance threshold 0.05, BASS-BASR (HK-RR) and XDHA-XDHC have significant coevolution, while LOLC-MACA and ACRE-ENVR do not. This is in good agreement with the fact that BASS-BASR and XDHA-XDHC possess known direct physical interactions, while LOLC-MACA and ACRE-ENVR do not. Surprisingly, MALG-MALK has a large pMirrortree score while possessing a known direct physical interaction, but this is mitigated by the fact that the close paralogs POTI-POTA have significant coevolution according to their pMirrortree score.

References cited in S1 Table

1. F. Pazos and A. Valencia. Similarity of phylogenetic trees as indicator of protein–protein interaction. *Protein Eng. Des. Sel.*, 14(9):609–614, 2001.

2. D. Ochoa and F. Pazos. Studying the co-evolution of protein families with the Mirrortree web server. *Bioinformatics*, 26(10):1370–1371, `http://csbg.cnb.csic.es/mtserver`, May 2010.

3. D. Ochoa, D. Juan, A. Valencia, and F. Pazos. Detection of significant protein coevolution. *Bioinformatics*, 31(13):2166–2173, `http://csbg.cnb.csic.es/pMT/`, Jul 2015.
